# Comparison of prognosis of five scoring systems in emphysematous pyelonephritis patients requiring intensive care

**DOI:** 10.1007/s11255-023-03733-8

**Published:** 2023-08-09

**Authors:** Mokhtar Bibi, Kays Chaker, Yassine Ouanes, Ramla Baccouch, Mohamed Anouar Madani, Houssem Mediouni, Boutheina Mosbahi, Kheireddine Mourad Dali, Moez Rahoui, Yassine Nouira

**Affiliations:** 1 Department of Urology, La Rabta Hospital, 1006 Tunis, Tunisia; 2 Department of Emergency, La Rabta Hospital, Tunis, Tunisia; 3 Department of Anesthesiology, La Rabta Hospital, Tunis, Tunisia

**Keywords:** Emphysematous pyelonephritis, Sepsis, Complications, Mortality, Prognosis, Scoring system

## Abstract

**Introduction:**

Our study aimed to evaluate the performance of Quick Sepsis-related Organ Failure Assessment (qSOFA), Modified Early Warning Score (MEWS), National Early Warning Score (NEWS), Systemic Inflammatory Response Syndrome (SIRS), and Global Research in the Emphysematous Pyelonephritis group (GREMP) in predicting the need of admission in intensive care units (ICU) for emphysematous pyelonephritis (EPN) patient.

**Patients and methods:**

In this retrospective study, we reviewed 70 patients admitted to our department from January 2008 to October 2022. Data on clinical presentation and EPN management were noted. The five scoring systems were calculated by one investigator. Univariate and multivariate analyses were used to assess predictive factors of severe sepsis and mortality. Statistical analysis was made using SPSS version 22.

**Results:**

Mean age was 61.83 years with 65.7% diabetes. As per Huang and Tseng classification, 41 patients had class I EPN, 7 had class II EPN, 8 had class IIIa, 6 class IIIB EPN, and 8 had class IV EPN. Seventeen patients (24.28%) were admitted to ICU with an 18.57 mortality rate. Univariate analysis showed that ICU admission was significantly associated with higher respiration rate and heart rate, lower systolic blood pressure, confusion, CRP, lactate and creatinine serum (*p* = 0.0001, *p* = 0.0001, *p* = 0.001, *p* = 0.007, *p* = 0.004, *p* = 0.001, *p* = 0.001, respectively). All five scores and Huang and Tseng classification were significantly predictive of admission to ICU. All five scores showed good results under the area curves to predict ICU entry with 0.915, 0.895, 0.968, 0.887, and 0.846 for qSOFA, MEWS score, NEWS score, SIRS, and GREMP score, respectively.

**Conclusion:**

NEWS score seemed to be the best performing physiologic score among the five scoring systems studied and may help with biological and radiological findings to quickly identify EPN patients that need intensive care unit.

## Introduction

Emphysematous pyelonephritis (EPN) is a fatal disease that is characterized by acute necrotizing parenchymal and perirenal infection [1]. The first case of presence of gas due to renal infection was reported in 1898 by Kelly and MacCallum [1]. Schultz and Klorfein recognized EPN as a condition that combines gas formation in the kidney with acute renal infection [2]. Pathogenetic mechanisms of EPN include high glucose levels in tissues, presence of gas-producing organisms, impaired host immunity, impaired tissue perfusion and urinary tract obstruction [3]. Historically, early nephrectomy or surgical drainage was the standard of care but mortality rate was 50% [3–5]. Mortality dropped to 13% with conservative management due to advent of advanced antibiotics, percutaneous or endourological drainage and CT scan findings [6–9]. A recent meta-analysis demonstrates that significant risk factors for mortality in EPN are sepsis, shock, and disturbance of consciousness with OR between 12 and 15 [10]. Therefore, it is crucial to quickly identify patients who need admission to intensive care units (ICU) to improve EPN prognosis. Quick Sequential Organ Failure Assessment Score (qSOFA), Modified Early Warning Score (MEWS), National Early Warning Score (NEWS), and Systemic Inflammatory Response Syndrome (SIRS) are among the clinical scoring systems used to predict patient outcomes in emergency care [11–14]. Recently, a multicenter international study reviewed 570 patients from 15 centers and proposed the GREMP score to assess EPN mortality [15]. Few studies compared GREMP score with physiologic scoring systems for EPN patients who need ICU. Our study aimed to assess which of the five scoring systems was best at predicting EPN patients requiring ICU admission.

## Patients and methods

### Study design and participants

This retrospective single-center study was conducted in our tertiary hospital. Patients with a clinico-radiological diagnosis of EPN between 2008 and 2022 were included. We excluded patients with fistulous connections between the urinary and intestinal tracts or a history of endourological procedures within 1 month. The study was approved by the ethics committee of our institution.

### Variables, scoring systems, and definitions

Patients’ data, including demographics, laboratory investigations, and clinical outcomes, were extracted from paper-based medical records. For each patient, qSOFA, MEWS, NEWS, SIRS, and GREMP scores were calculated.

EPN is a necrotic infection of the kidney and CT scan showed presence of gas within urinary tract or renal parenchyma or perirenal spaces. EPN CT scan classification includes class 1: gas in the collecting system only; class 2: gas in the renal parenchyma without extension to extra-renal space; (3) class 3A: extension of gas or abscess to perinephric space; class 3B: extension of gas or abscess to pararenal space; and class 4: bilateral or solitary kidney with EPN according to Huang and Tseng classification [7]. The general criteria for ICU admission for EPN patients included support of at least two organ systems or advanced respiratory support.

### Statistical analysis

We used IBM SPSS interpretation 22 software for statistical analysis. Descriptive data were presented as mean and standard deviation (SD) for typically distributed continuous variables, and as median and interquartile range (IQR) for disposed variables. Data were presented as frequency and percentages (%) for categorical data. Comparison of proportions was performed using the ki-square test and non-parametric data by Mann–Whitney *U* test. All results were considered significant at a *p* value of < 0.05. Receiver operating characteristic curves were generated for five scores using logistic regression, to obtain the area under curve (AUC) value for comparison of ICU admission predictability. We used Youden’s index to identify optimal cutoff and we calculated sensitivity and specificity of each score to predict admission to ICU.

## Results

**Fig. 1:**
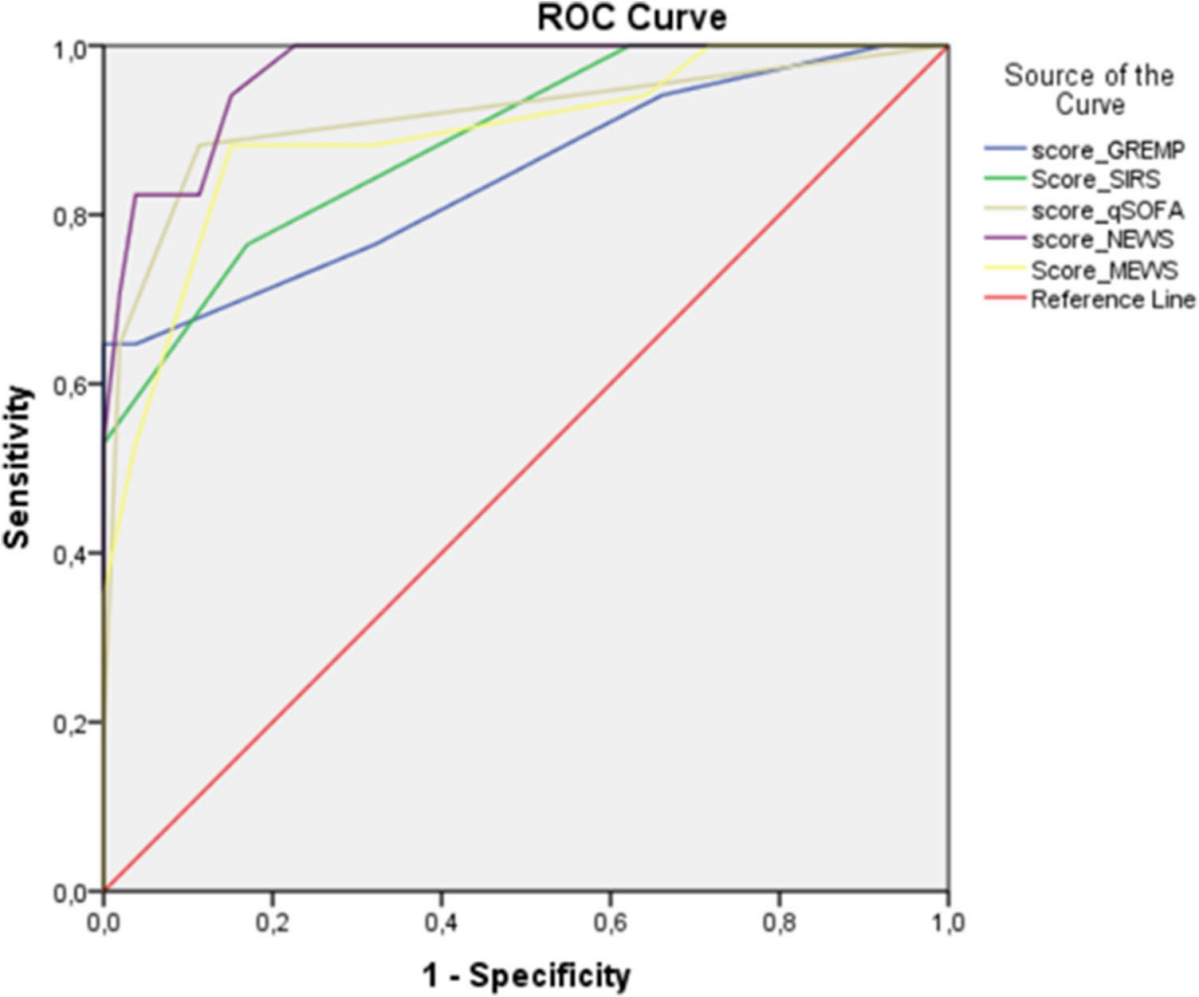
Receiver operating curves for predicting intensive care units admission according five scores

**Fig. 2:**
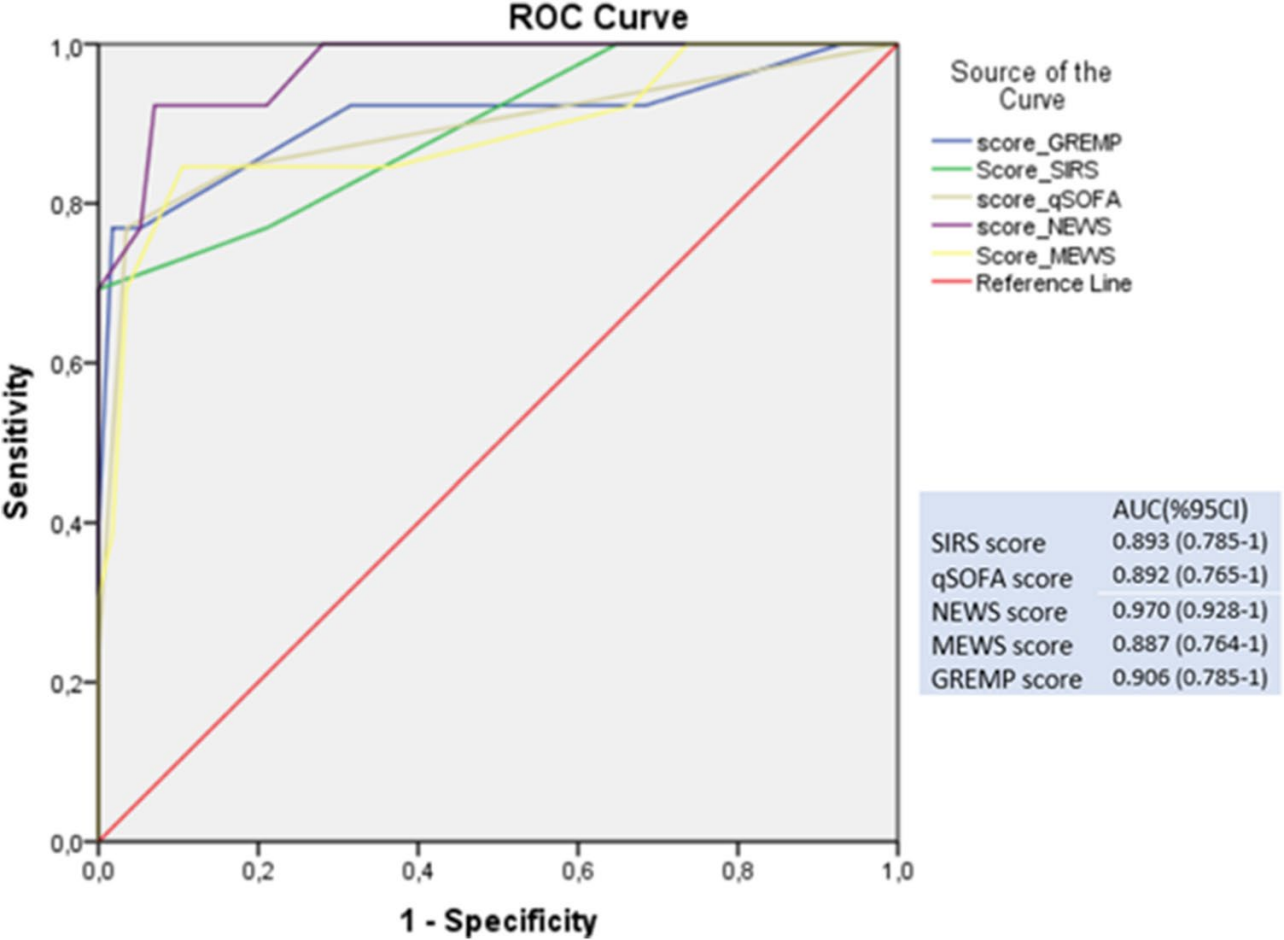
Receiver operating curves for predicting mortality according to five scores

A total of 70 patients were included with a mean age of 61.83 ± 14.11 years. More than 65% of patients were women with diabetes. Seventeen patients (24.28%) were admitted to ICU. Mortality rate was 18.57%. The leading microorganism of cultures from urine (*n* = 38), blood (*n* = 15) was *Escherichia coli* then *Klebsiella pneumonia*. In CT Huang and Tseng classification, 41 patients had class I EPN, 7 had class II EPN, 8 had class IIIa, 6 had class IIIB EPN, and 8 had class IV EPN. Conservative management with early drainage was indicated in 77.14% of cases. Baseline characteristics of EPN patients and microbiology are shown in Tables 1 and 2. Compared to patients who did not require reanimation, patients in ICU unit presented higher respiration rate and heart rate (*p* = 0.0001), lower systolic blood pressure (*p* = 0.0001), lower GCS score (*p* = 0.007), lower prothrombin time (*p* = 0.0001), higher creatinine (*p* = 0.0001), and higher CT scan Huang and Tseng classification (*p* = 0.0001). All clinical parameters were mostly included in five scores. Univariate analysis showed that all five scores were significantly higher for patients admitted to ICU (*p* = 0.0001) (Table 3). AUROC analysis demonstrated the predictability of the five scoring systems, listed in descending order: NEWS 0.968; qSOFA 0.915; MEWS 0.895; SIRS score 0.887; and GREMP score 0.846 (Table 4 and Fig. 1). All five scores performed well in EPN patients, each achieving AUC > 0.7. Among five scores, NEWS was best at predicting the need for ICU admission, with a sensitivity of 94.11%, specificity of 84.9%, positive predictive value of 66.66%, and negative predictive value of 97.82% (Table 4). Regarding mortality risk, AUROC results of five scores were excellent listed in descending order: NEWS 0.970; GREMP 0.906; SIRS 0.893; qSOFA 0.892; and MEWS 0.887 (Fig. 2).

**Table 1:** Characteristics of emphysematous pyelonephritis patients

	All	Admission to ICU	No need to ICU	*p*
Patients, *n*	70	*n* = 17	*n* = 53	
Age, years (mean ± SD)	61.83 ± 14.11	60.94 ± 13.34	62.11 ± 14.45	0.76*
Gender, *n* (%)				
Man	21 (30)	6 (35.3)	15 (28.3)	0.72*
Women	49 (70)	11 (64.7)	38 (71.7)	
Diabetes, *n* (%)				
No	24 (34.3)	6 (35.3)	18 (34)	0.57*
Yes	46 (65.7)	11 (64.7)	35 (66)	
Vital sign				
Temp, median (IQR)	38.5 (38–39)	38.5 (37.85–39)	38.5 (38–39)	0.95
Respiration rate	16 (14–20)	22 (20.25–24.75)	15 (13–18)	0.0001
Heart rate	95 (85–110)	116 (110–128.75)	88.5 (84.25–97.25)	0.0001
Systolic BP	117 (106–136)	93.5 (84.25–104)	127 (116–137)	0.0001
Glasgow score, *n* (%)				
≤ 8	1 (1.4)	1 (5.9)	0	0.007*
9–11	6 (8.6)	4 (23.5)	2 (3.8)	
≥ 12	63 (90)	12 (70.6)	51 (96.2)	
Laboratory results				
WBC (10^3^ counts/mm^3^)	17,689.76 ± 9535	20,034.71 ± 12,124.9	16,937.6 ± 8545	0.24*
Platelet (10^3^ counts/mm^3^)	217,028.57 ± 140,909	170,470.59 ± 151,622	231,962 ± 135,414	0.11*
Prothrombin time (%)	73 (55–85)	45 (36.5–55.75)	79.5 (66–86.75)	0.0001
Creatinine (mmol/l)	149.5 (89.75–249.23)	276.36 (152.25–643.31)	120.38 (89–191.75)	0.0001
CRP (mg/dL)	232.66 ± 107.54	296.69 ± 82.3	212.12 ± 107.19	0.004*
Na (mEq/L)	132 (129–137)	131 (127.5–135.5)	132 (130–137)	0.19
Lactate (mg/dL)	2 (1–3)	4 (3.45–5.15)	1 (1–2)	0.0001
Glucose (mg/dL)	2.5 (1.8–3.42)	3.55 (1.85–4)	2.43 (1.64–3)	0.094
pH	7.36 (7.26–7.40)	7.25 (7.15–7.29)	7.39 (7.32–7.40)	0.0001
PaCO2 (mmHg)	32 (26–35)	23(18.5–27.5)	32.5 (30–36)	0.0001
HCO3 − (mmol)	19 (12.75–21)	10 (7.23–14.75)	20 (16.25–22)	0.0001
PaO2 (mmHg)	88 (73–98)	66.5 (56–87.5)	95 (75–99)	0.0001
Radiologic results				
Urolithiasis *n* (%)	62 (88.6)	15 (88.2)	47 (88.7)	0.92*
Huang–Tseng CT				
Class I	41 (58.6)	4 (23.5)	37 (69.8)	0.0001*
Class II	7 (10)	2 (11.8)	5 (9.4)	
Class IIIA	8 (11.4)	1 (5.9)	7 (13.2)	
Class IIIB	6 (8.6)	4 (23.5)	2 (3.8)	
Class IV	8 (11.4)	6 (35.3)	2 (3.8)	
Management				
Antimicrobials, *n* (%)				
C3G + aminoglycosides	42 (60)	4 (23.5)	38 (71.1)	0.001*
FQ + aminoglycosides	3 (4.3)	0	3 (5.7)	
Carbapenems + aminoglycoside	21 (30)	11 (64.7)	10 (18.9)	
Piperacillin + tazobactam	4 (5.7)	2 (11.8)	2 (3.8)	
Ureteral stent	43 (61.4)	5 (29.4)	38 (71.7)	0.008*
Nephrostomy	11 (15.7)	5 (29.4)	6 (11.3)	
Nephrectomy	16 (22.9)	7 (41.2)	9 (17)	
Mortality rate	13 (18.57)	12 (70.6)	1 (1.9)	0.0001*

*Temp* temperature, *ESBL* extended spectrum beta-lactamase microorganism, *BP* blood pressure, *WBC* white blood cell, *CRP* C-reactive protein, *Na* sodium, *IQR* interquartile range (25%–75%)

*Student test or *χ*^2^ tests

**Table 2:** Microbiology of emphysematous pyelonephritis

Bacteria/culture	Urine (*n* = 53)	Blood (*n* = 24)
*Escherichia coli*	38 (71.69)	15 (62.5)
*Klebsiella pneumonia*	8 (15.09)	5 (20.83)
*Pseudomonas aeruginosa*	2 (3.77)	3 (12.5)
*Proteus mirabilis*	3 (5.66)	1 (4.16)
*Enterococcus faecalis*	2 (3.77)	0

**Table 3:** Scoring systems for predicting the clinical outcomes of emphysematous pyelonephritis

	All	Admission to ICU	No need to ICU	*p *value
	(*n* = 70)	(*n* = 17)	(*n* = 53)	
SIRS score(median, IQR)	2 (1–3)	3.5 (2.25–4)	2 (1–2)	0.0001
qSOFA score	0.5 (0–1)	2 (1–2)	0 (0–1)	0.0001
NEWS score	3 (1–6)	8.5 (7–11.75)	2 (1–3)	0.0001
MEWS score	2 (1–4.25)	5.5 (4–7.75)	2 (0–3)	0.0001
GREMP score	2(1–3)	5(2.5–6)	2(1–3)	0.0001

*IQR* interquartile range (25%–75%), *SIRS* systemic inflammatory response syndrome, *ICU* intensive care units

**Table 4:** Accuracies, sensitivities, specificities, and, predictive values for SIRS, qSOFA, NEWS, MEWS, and GREMP scores in predicting intensive care units admission

	Optimal cut-\off	AUC (%95 CI)	Sensitivity%	Specificity%	PPV%	NPV%
SIRS score	3	0.887 (0.795–0.97)	76.5	83	59.09	91.66
qSOFA score	1	0.915 (0.817–0.93)	88.23	88.67	71.42	95.91
NEWS score	5	0.968 (0.933–0.95)	94.11	84.90	66.66	97.82
MEWS score	4	0.895 (0.89–0.93)	88.23	84.90	65.21	95.74
GREMP score	3	0.846 (0.722–0.97)	76.47	67.92	43.33	90

*AUC* area under curve, *CI* confidential interval, *PPV* positive predictive value, *NPV* negative predictive value

## Discussion

This study showed that EPN remains a life-threatening renal infection with 18.57% mortality. Early identification of patients at high risk of death is the key to reducing mortality from EPN. In our study, risk factors of poor prognosis were hemodynamic instability, respiration failure, shock, thrombocytopenia, confusion, and Huang–Tseng classification. These factors were significant predictive of mortality in two recent meta-analysis about EPN management [10, 16]. Also, it has been confirmed that mortality significantly increased with delayed ICU admission for critically ill surgical patients [17]. Risk factors as well as scoring systems should be part of the decision on the most effective treatment. In our study, NEWS score was the best scoring system to predict admission to ICU for EPN patients with sensitivity of 94.11% and specificity of 84.9%. Except temperature, all clinical parameters of NEWS score were significantly predictive for admission to ICU. NEWS is an early warning score designed by the National Early Warning Score Development and Implementation Group (NEWSDIG) for the Royal College of Physicians to identify who need urgent care [13]. NEWS performed equally well, or better, for surgical as for medical patients. For death within 24 h, the AUROC for surgical and medical admissions were 0·914 and 0.902, respectively [18]. Previous studies have also found NEWS to be equal or superior to qSOFA, Systemic Inflammatory Response Syndrome score for predicting hospital mortality and ICU admission in emergency admissions treated as sepsis [19, 20]. Yap et al. demonstrated that the NEWS score predicted intensive care admission in a cohort of 65 patients with EPN (AUC = 0.825) [21]. The author suggested that EPN patients with a NEWS of the cutoff point 3 and below were unlikely to require ICU admission (NPV 96.55%) [21]. In our study, NEWS cutoff point was 5 with NPV at 97.82%. Chawla et al. reviewed 90 EPN patients and found that NEWS score was the best at predicting the need for ICU admission. Like in our study, the author found that NEWS score ≥ 5 was the best predictor of the need for urgent admission to the ICU [22]. In terms of predicting EPN mortality, Krishnamoorthy et al. prospectively reviewed 131 EPN patients in 2019 and proposed a prognostic scoring system to predict mortality including 18 parameters [23]. Patients were stratified based on the mortality risk into the very low, low, intermediate, and high-risk groups [23]. GREMP score is a simple tool to assess EPN mortality with AUC = 0.90 [13]. This score included four biological parameters, pararenal gas extension on CT scan and quick SOFA ≥ 2 [13]. In our study, NEWS and GREMP predicted EPN mortality with AUC of 0.970 and 0.906, respectively. The main limitation was a retrospective single-center study with limited size of EPN population. Also, patients’ records were collected over a decade with different EPN management and outcomes.

## Conclusion

NEWS seemed a simple and objective tool for the evaluation of need for ICU admission in EPN patients presenting to the ED. CT scan provide information of the extent and localization of the EPN and helps us to decide conservative management or early nephrectomy.
